# A Variable Step Crow Search Algorithm and Its Application in Function Problems

**DOI:** 10.3390/biomimetics8050395

**Published:** 2023-08-28

**Authors:** Yuqi Fan, Huimin Yang, Yaping Wang, Zunshan Xu, Daoxiang Lu

**Affiliations:** Key Laboratory of Advanced Manufacturing and Intelligent Technology, Ministry of Education, School of Mechanical and Power Engineering, Harbin University of Science and Technology, Harbin 150080, China

**Keywords:** crow search algorithm, optimization algorithm, test function

## Abstract

Optimization algorithms are popular to solve different problems in many fields, and are inspired by natural principles, animal living habits, plant pollinations, chemistry principles, and physic principles. Optimization algorithm performances will directly impact on solving accuracy. The Crow Search Algorithm (CSA) is a simple and efficient algorithm inspired by the natural behaviors of crows. However, the flight length of CSA is a fixed value, which makes the algorithm fall into the local optimum, severely limiting the algorithm solving ability. To solve this problem, this paper proposes a Variable Step Crow Search Algorithm (VSCSA). The proposed algorithm uses the cosine function to enhance CSA searching abilities, which greatly improves both the solution quality of the population and the convergence speed. In the update phase, the VSCSA increases population diversities and enhances the global searching ability of the basic CSA. The experiment used 14 test functions,2017 CEC functions, and engineering application problems to compare VSCSA with different algorithms. The experiment results showed that VSCSA performs better in fitness values, iteration curves, box plots, searching paths, and the Wilcoxon test results, which indicates that VSCSA has strong competitiveness and sufficient superiority. The VSCSA has outstanding performances in various test functions and the searching accuracy has been greatly improved.

## 1. Introduction

The optimization is to give existing solutions and parameters to present a satisfactory answer for a certain problem. For quite some time, people have conducted large research on various optimization problems. Newton and Leibniz founded calculus which can solve some optimization problems. Then, different mathematical concepts have been proposed, such as the steepest descent method and the linear programming solution method, which can be used in many fields [1,2,3].

For specific problems, traditional methods have produced specific optimization methods for different problems. However, most of these methods have specific requirements for the searching space which requires objective functions to be convex, continuously differentiable, and differentiable. These weaknesses of traditional optimization methods are limited in solving many practical problems [4,5,6,7]. These practical production problems have large-scale, non-linear, multi-extreme values, characteristics of multiple constraints, and non-convexities, making traditional optimization methods difficult to conduct mathematical modeling. Therefore, exploring information processing methods with intelligent features is valuable.

In practical applications, intelligent algorithms generally do not require problem special information, the constraint on the problem, the continuity, the differentiability, the convexity of the objective function, and the analytical expression. Intelligent algorithms have strong adaptability to uncertainty data in the calculation process. At present, intelligence algorithms mainly include African Vultures Optimization Algorithm (AVOA) [8], Beluga Whale Optimization (BWO) [9], Whale Optimization Algorithm (WOA) [10], Flow Direction Algorithm (FDA) [11], Grey Wolf Optimizer (GWO) [12], Harris Hawks Optimizer (HHO) [13], Sine Cosine Algorithm (SCA) [14], Spotted Hyena Optimizer (SHO) [15], Slime Mould Algorithm (SMA) [16], Symbiotic Organisms Search (SOS) [17], Wild Horse Optimizer (WHO) [18], Geometric Mean Optimizer (GMO) [19], Golden Jackal Optimization algorithm (GJO) [20], Coati Optimization Algorithm (COA) [21], Dandelion Optimizer (DO) [22], Remora Optimization Algorithm (ROA) [23], Great Wall Construction Algorithm (GWCA) [24], Generalized Normal Distribution Optimization (GNDO) [25], Pelican Optimization Algorithm (POA) [26], and so on [27,28,29,30].

These algorithms have been achieved in various engineering fields [31,32,33,34,35,36]. For solving large-scale optimization problems, intelligent algorithms are significantly superior to traditional mathematical programming methods in terms of computational times and complexities.

Crow Search Algorithm (CSA) was proposed by Alireza Askarzadeh in 2016 [37]. Crows will hide their food and remember its hiding location for several months. At the same time, they will track other crows to steal food. Based on the living habits of crows in nature, the crow search algorithm has been proposed. From the algorithmic perspective, the overall flying area of the crow population is the searching space. The position of each crow represents the algorithm feasible solution and the location of crow hidden food represents the algorithm’s objective function value. The best food position in the algorithm is the optimal solution in the searching space.

Shalini Shekhawat and Akash Saxena designed the Intelligent Crow Search Algorithm (ICSA) and used ICSA in the structural design problem, frequency wave synthesis problem, and Model Order Reduction [38]. Yilin Chen et al. introduced a robust adaptive hierarchical learning Crow Search Algorithm for feature selection [39]. Primitivo Díaz et al. introduced an improved Crow Search Algorithm Applied to Energy Problems [40]. Amrit Kaur Bhullar et al. proposed the enhanced crow search algorithm for AVR optimization [41]. Thippa Reddy Gadekallu et al. used CNN-CNS for handing gesture classification [42]. Malik Braik et al. designed a hybrid crow search algorithm for solving numerical and constrained global optimization problems [43]. Behrouz Samieiyan et al. applied Promoted Crow Search Algorithm (PCSA) to solve dimension reduction problems [44]. Qingbiao Guo et al. used an improved crow search algorithm for the parameter inversion of the probability integral method [45]. CSA has been applied in many fields.

In the basic CSA, crows update their positions by the fixed flight length in the searching space, wherein the fixed flight length will make the individual jump out of the fitness solution region, which can cause low searching accuracy. As a result, this paper proposes a variable step crow search algorithm (VSCSA). VSCSA uses Cosine function steps to update its positions. The rest of this paper is organized as follows: In Section 2, this paper gives the basic CSA. In Section 3, this paper proposes VSCSA. In Section 4, the function experiment results analysis is shown. In Section 5, the CEC2017 function experiment results analysis is shown. In Section 6, engineering application problems are shown. In Section 7, the conclusion is given.

## 2. Crow Search Algorithm

The crow is the general name of passerine corvus that is a large songbird which has a sturdy mouth and feet. Nostrils are circular and usually covered by feather whiskers. Crows like to live in groups and have strong clustering. They are forest and grassland birds with a steady gait. Except for a few species, they often gather in groups and nest, and wander in mixed groups during the autumn and winter seasons, flying and singing. Generally, the personality is fierce and full of aggressive habits. CSA is a metaheuristic algorithm based on crow intelligent behaviors. Crows will steal food by observing where the other birds hide their food, if a crow finds the thief, it will move to hiding places to avoid being a future victim. And crows use their own experiences to predict the pilferer’s behavior. In CSA, the crow overall flight area is the searching space, and the position of each crow gives a feasible solution. The crow hidden food represents the quality of the algorithm function value.

The CSA Step is given in this section.

Step 1: Initialize the problem and adjustable parameters.

Set CSA size *N*, the maximum number of iterations *iter_max_*, the flight length (*fl*), the awareness probability *AP*, and the searching dimension is *d*. The crow *i* at one iteration in the searching space is specified by a vector *x^i,iter^*(*i* = 1, …, *N*; *iter* = 1, …, *iter_max_*). The searching upper bound is *ub_i_*(*i* = 1, …, *N*) and the searching lower bound is *lb_i_*(*i* = 1, …, *N*),

Step 2: Initialize position and memory.

Each crow will save its hidden food location m during each iteration, which represents the best position the crow currently has because during the initial iteration of the algorithm, the crow is inexperienced. Therefore, the initial memory, which is the location where the crow first hides its food, is set as their initial position.

(1) $$Crows=\left[\begin{array}{cccc} x_{1}^{1} & x_{2}^{1} & \cdots & x_{d}^{1}\\ x_{1}^{2} & x_{2}^{2} & \cdots & x_{d}^{2}\\ \vdots & \vdots & \vdots & \vdots \\ x_{1}^{N} & x_{2}^{N} & \cdots & x_{d}^{N} \end{array}\right]$$

(2) $$Memory=\left[\begin{array}{cccc} m_{1}^{1} & m_{2}^{1} & \cdots & m_{d}^{1}\\ m_{1}^{2} & m_{2}^{2} & \cdots & m_{d}^{2}\\ \vdots & \vdots & \vdots & \vdots \\ m_{1}^{N} & m_{2}^{N} & \cdots & m_{d}^{N} \end{array}\right]$$

Step 3: Evaluate the objective function.

Compute one crow position.

Step 4: Generate a new position.

Crow *i* will generate a new position. In this case, two states will happen:

State 1: Crow *i* will approach crow *j*.

State 2: Crow *j* will go to another position.

States 1 and 2 can be expressed as follows:

(3) $$x^{i,iter+1}=\left\{ \begin{array}{l} x^{i,iter}+r_{i}\times fl^{i,iter}\times \left(m^{j,iter}-x^{i,iter}\right)\begin{array}{cc} & r_{j.iter}\geq AP^{j.iter} \end{array}\\ \begin{array}{ccc} a & random & position\begin{array}{cc} \begin{array}{cc} & otherwise \end{array} & \end{array} \end{array} \end{array}\right.$$

where *r_i_* is a random number in the range of [0, 1] and *fl^i;iter^* denotes the flight length of crow *i* at iteration iter. AP denotes the awareness probability.

Step 5: Check the feasibility of new positions.

Check the new position feasibility of each crow.

Step 6: Evaluate fitness functions of new positions.

Calculate all feasible solutions. The function value for the new position of each crow will be calculated.

Step 7: Update memory

The crows update their memory as follows:

(4) $$m^{i,iter+1}=\left\{ \begin{array}{l} \begin{array}{cc} x^{i,iter+1} & f\left(x^{i,iter+1}\right)\begin{array}{ccc} & is & \begin{array}{cc} better & than\begin{array}{cc} & f\left(m^{i,iter}\right) \end{array} \end{array} \end{array} \end{array}\\ \begin{array}{cc} m^{i,iter} & otherwise \end{array} \end{array}\right.$$

Compare all fitness function values. If there is a better fitness function value of the new position, the memory will be updated.

Step 8: Check termination criterion.

Calculate *iter* = *iter* + 1. Stop if the termination criterion is met *iter* = *iter_max_*. If not, Steps 4–7 are repeated until the *iter_max_* is reached.

## 3. Variable Step Crow Search Algorithm

In the original CSA, crows constantly update their positions in the searching space, but their flight length fl is fixed, and the solutions to the search problem are diverse. When initializing a population, individuals often cannot directly locate the optimal solution and approach the region where the optimal solution is located without prior exploration experience in the searching space as a guide. Therefore, the searching process should be carried out in multiple different directions to expand the searching scope and thereby increase the probability of approaching the area. In addition, individuals in the population often wish to visit unexplored areas when exploring the searching space, thereby increasing the breadth of the search. And from the CSA position update formula, it can be seen that the crow population mainly updates its position by moving towards a fixed flight length. Therefore, as the species iteration continues, the crow population will gradually cluster and the population diversity will gradually decrease, which can easily lead to a single searching direction and form too many local optima which is not conducive to the algorithm’s small-scale search in the later stage. To solve this problem, this article proposes a variable step crow search algorithm (VSCSA).

The cosine function is a Periodic function with a minimum positive period of 2π. When the independent variable is an integer 2kπ (k is an integer), the function has a maximum value of 1. When the independent variable is (2k + 1) π (k is an integer), the function has a minimum value of −1. The cosine function is an even function, and its image is symmetric about the *y*-axis.

(5) $$x_{new}^{i,iter+}{}^{1}=x_{new}^{i,iter}+\left|\cos\left(r_{i}\right)\right|\times \left(m^{j,iter}-x_{new}^{i,iter}\right)\begin{array}{cc} & r_{j.iter}\geq AP^{j.iter} \end{array}$$

When crow *j* knows that crow *i* is following it, then as a result, crow *j* will go to another position by the searching upper bound.

(6) $$x_{new}^{i,iter+}{}^{1}=0.5\times \left(ub_{i}\times rs_{i}+m^{j,iter}\right)$$

New states 1 and 2 can be expressed as follows:

(7) $$x_{new}^{i,iter+}{}^{1}=\left\{ \begin{array}{l} x_{new}^{i,iter}+\left|\cos\left(r_{i}\right)\right|\times \left(m^{j,iter}-x_{new}^{i,iter}\right)\begin{array}{cc} & r_{j.iter}\geq AP^{j.iter} \end{array}\\ \begin{array}{ccc} 0.5\times \left(ub_{i}\times rs_{i}+m^{j,iter}\right) & & \begin{array}{cc} \begin{array}{cc} & otherwise \end{array} & \end{array} \end{array} \end{array}\right.$$

where *rs_i_* is a random number in the range of [−1, 1] and *ub_i_*(*i* = 1, …, *N*) is the searching upper bound.

VSCSA improves the population diversity and the changing pattern search guidance method during the evolution process. In the early searching stage, the population diversity is relatively high. Cosine steps serve as a guide for population evolution, which can avoid blind individual searching and population diversity rapid decay. This meets the requirement that the algorithm should conduct a large-scale exploration as much as possible during the initial iteration. In the later searching stages, the proposed algorithm will shift from a global exploration to a local development, which can avoid the divergence in search directions. When the population falls into the local optima, the proposed algorithm can use individuals generated by cosine steps as searching guides to effectively increase population diversities and jump out of different local optima areas. Therefore, the proposed strategy reflects the adaptive interaction between population diversities and multiple search guided individuals, the changes in the population searching steps reflect different stages of evolution, and different searching guided methods can be adaptively selected. In turn, different guided methods can alter the diversity of the population, expand the algorithm searching range, and strengthen the algorithm searching precision.

**Figure 1:**
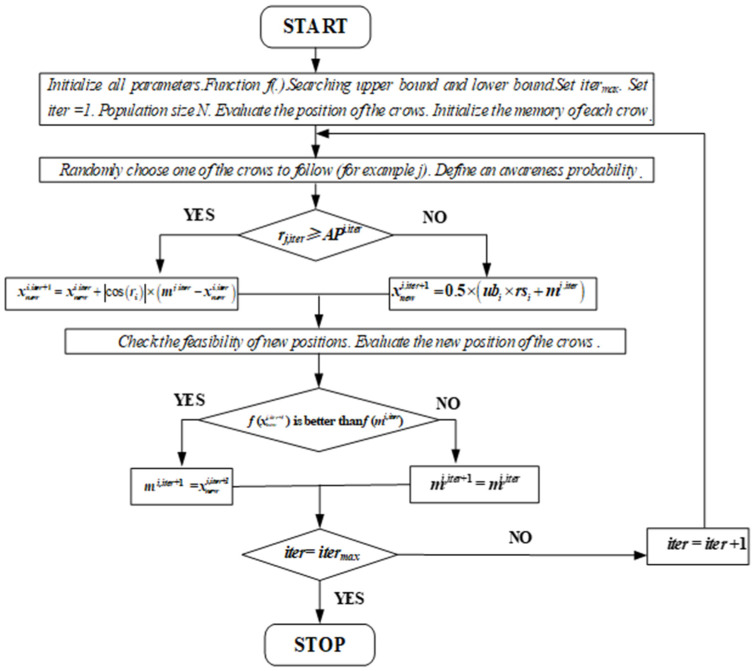
The VSCSA Flowchart.

The VSCSA Flowchart can be presented in Figure 1 as follows:

The VSCSA pseudo code can be summarized in Algorithm 1.
**Algorithm** **1:** VSCSA.**Input:** *Function f(.). Searching upper bound and lower bound. Set iter_max_. Set iter = 1. Population size N. Evaluate the position of the crows. Initialize the memory of each crow.* **While (***iter < iter_max_***)** **For** *i = 1:N* *Randomly choose one of the crows to follow (for example j).* *Define an awareness probability.* **If 1** *r_j,iter_≥ AP^i.iter^* $x_{new}^{i,iter+1}$ = $x_{new}^{i,iter}$+ |cos(*r_i_*)| × (*m^j,iter^* − $x_{new}^{i,iter}$) ***Else*** $x_{new}^{i,iter+1}$ = 0.5 × (*ub_i_* × *rs_i_* + *m^j,iter^*) ***End If 1*** ***End For*** *Check the feasibility of new positions.* *Evaluate the new position of the crows.* *Update the memory of crows:* ***If 2***
*f* ($x_{new}^{i,iter+1}$) is better than *f* (*m^i,iter^*). *m^i,iter+1^* = $x_{new}^{i,iter+1}$ ***Else*** *m^i,iter+1^ = m^i,iter^* ***End If 2*** *iter = iter + 1* ***End While***

## 4. Function Experiment Results

### 4.1. Experiment Environments

Different functions are in Table 1. In Table 1, *D* is the searching dimension and *f*_min_ is the idea function value. Range is the searching scope. Different optimal solutions of high-dimensional testing functions in this paper are hidden in a smooth and narrow parabolic valley, with broad searching space, tall obstacles, and a large number of local minimum points. This paper uses different test functions for comparing VSCSA and standard CSA performances. This paper tests VSCSA with the cuckoo search algorithm (CS) [46], the sine cosine algorithm (SCA) [14], and the moth-flame optimization algorithm (MFO) [47]. CS was proposed by Xin-She Yang and was inspired by the cuckoo incubation mechanism in nature. The size of the cuckoo bird is similar to that of a pigeon, but it is slender and has a dark gray upper body. SCA, which was proposed by Seyedali Mirjalili in 2016, was inspired by sine and cosine mathematical terminology. MFO, which was proposed by Seyedali Mirjalili in 2015, was inspired by the moth navigation in nature called the transverse orientation. In this chapter, the CS discovery probability was set as 0.25 and the step was set as 0.25. For SCA, *a* = 2, *r*_2_ = 2*π*, *r*_3_, and *r*_4_ were selected in [0, 1]. In CSA, *fl* = 2. All algorithm parameters were selected from the original algorithm literature. The population size was 20, the maximum iterations were 400, and it was ran 10 times in MATLAB (R2016b).

### 4.2. Data Results

In Table 2 and Table 3, Min, Max, Ave, and Var mean the minimum value, the maximum value, the average value, and the variance deviation. Table 2 shows two-dimension function results. Table 3 shows high-dimension functions results. For two-dimension functions, VSCSA can obtain the ideal function values in *f*_2_ to *f*_5_, *f*_12(*D*=2)_, and *f*_13(*D*=2)_. And VSCSA can obtain the ideal values of all evaluation indexes in *f*_2_ to *f*_4_. CSA can obtain ideal function values in *f*_12(*D*=2)_, *f*_13(*D*=2)_. MFO can obtain the ideal function values in *f*_2_ to *f*_5_, *f*_12(*D*=2)_, and *f*_13(*D*=2)_. MFO can obtain the ideal values of all evaluation indexes in *f*_2_, *f*_3_, *f*_5_. SCA can obtain the ideal function values in *f*_2_ to *f*_4_, *f*_12(*D*=2)_, and *f*_13(*D*=2)_. SCA can obtain the ideal values of all evaluation indexes in *f*_2_ to *f*_4_. Min values of MFO in *f_1_*_0_ and *f*_14(*D*=2)_ are better than those of VSCSA. Min value of SCA in *f*_14(*D*=2)_ is better than that of VSCSA. For high dimension functions, the Min values of SCA in *f*_11(*D*=30)_, *f*_12(*D*=60)_, *f*_13(*D*=60)_, *f*_13(*D*=200)_ are better than those of VSCSA. Min value of MFO in *f*_12(*D*=30)_ is better than that of VSCSA. VSCSA in other results are all less than comparative algorithms. VSCSA can ensure continuous evolution and has good convergence speed and optimization accuracy. Especially for multi-peak high dimension functions with rotational characteristics, the proposed algorithm can better overcome the interference caused by local extreme points in the solving process, can prevent premature convergence, ensure continuous population evolution, and ultimately achieve a high optimization accuracy.

### 4.3. Iteration Results

**Figure 2:**
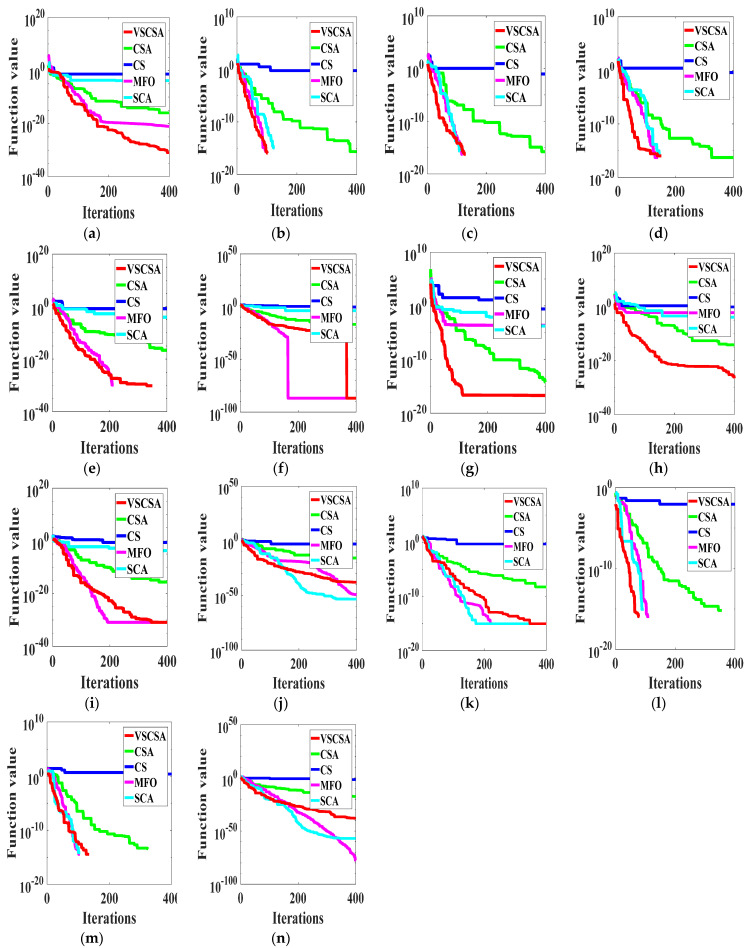
Iteration curves of two−dimension functions. (**a**) *f*_1_; (**b**) *f*_2_; (**c**) *f*_3_; (**d**) *f*_4_; (**e**) *f*_5_; (**f**) *f*_6_; (**g**) *f*_7_; (**h**) *f*_8_; (**i**) *f*_9_; (**j**) *f*_10_; (**k**) *f*_11(*D*=2)_; (**l**) *f*_12(*D*=2)_; (**m**) *f*_13(*D*=2)_; (**n**) *f*_14(*D*=2)_.

**Figure 3:**
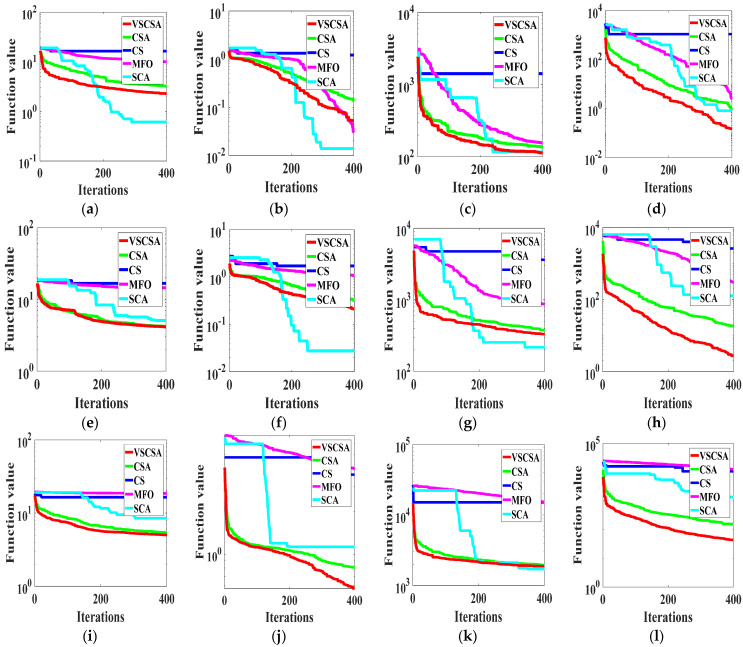
Iteration curves of high−dimension functions. (**a**) *f*_11(*D*=30)_; (**b**) *f*_12(*D*=30)_; (**c**) *f*_13(*D*=30)_; (**d**) *f*_14(*D*=30)_; (**e**) *f*_11(*D*=60)_; (**f**) *f*_12(*D*=60)_; (**g**) *f*_13(*D*=60)_; (**h**) *f*_14(*D*=60)_; (**i**) *f*_11(*D*=200)_; (**j**) *f*_12(*D*=200)_; (**k**) *f*_13(*D*=200)_; (**l**) *f*_14(*D=200*)_.

This paper gave algorithm optimal iteration curves after 10 independent operations, as shown in Figure 2 and Figure 3. Compared with different algorithms in two-dimension iteration curves, VSCSA has the fastest iteration curve except for *f*_10_, *f*_13(*D*=2)_, *f*_14(*D*=2)_. In *f*_10_, SCA has the fastest iteration curve. In *f*_13(D=2)_, *f*_14(*D*=2)_, MFO has the fastest iteration curve, and CSA has the second fast iteration curve. Compared with different algorithms in high-dimension iteration curves, VSCSA has the fastest iteration curve except *f*_11(*D*=30)_, *f*_12(*D*=30)_, *f*_12(*D*=60)_, *f*_13(*D*=60)_. In *f*_11(*D*=30)_, *f*_12(*D*=30)_, *f*_12(*D*=60)_, *f*_13(*D*=60)_, SCA has the fastest iteration curve. The VSCSA has outstanding performances in various test functions, whereby especially the searching accuracy has been greatly improved. Therefore, the iteration curve can display that VSCSA has a strong searching performance.

### 4.4. Box Plot Results

**Figure 4:**
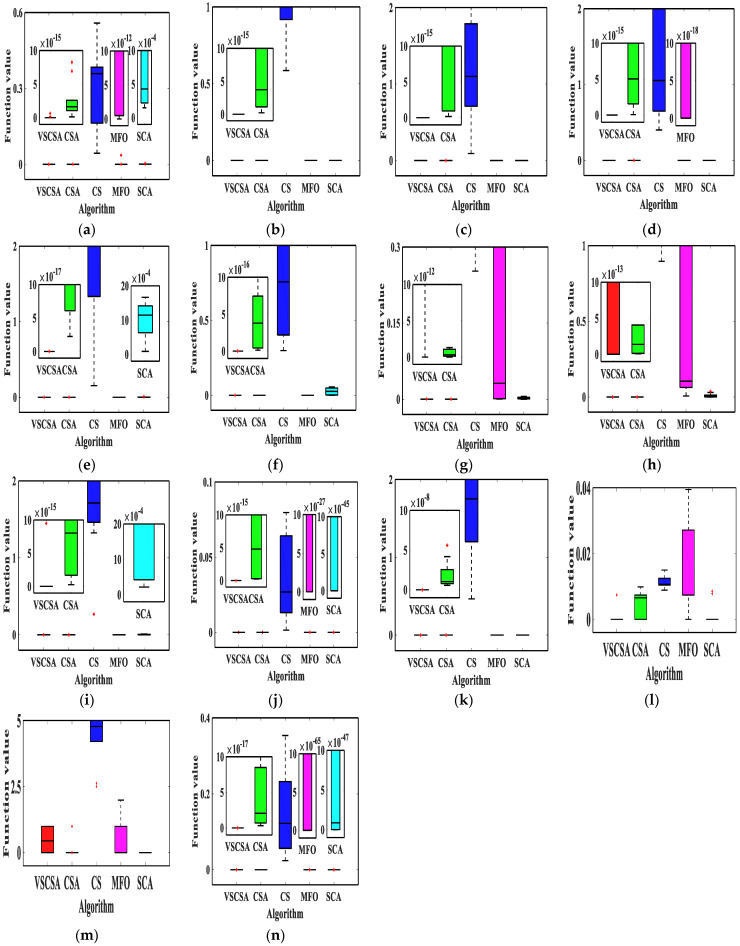
Box plot charts of two−dimension functions. (**a**) *f*_1_; (**b**) *f*_2_; (**c**) *f*_3_; (**d**) *f*_4_; (**e**) *f*_5_; (**f**) *f*_6_; (**g**) *f*_7_; (**h**) *f*_8_; (**i**) *f*_9_; (**j**) *f*_10_; (**k**) *f*_11(*D*=2)_; (**l**) *f*_12(*D*=2)_; (**m**) *f*_13(*D*=2)_; (**n**) *f*_14(*D*=2)_.

**Figure 5:**
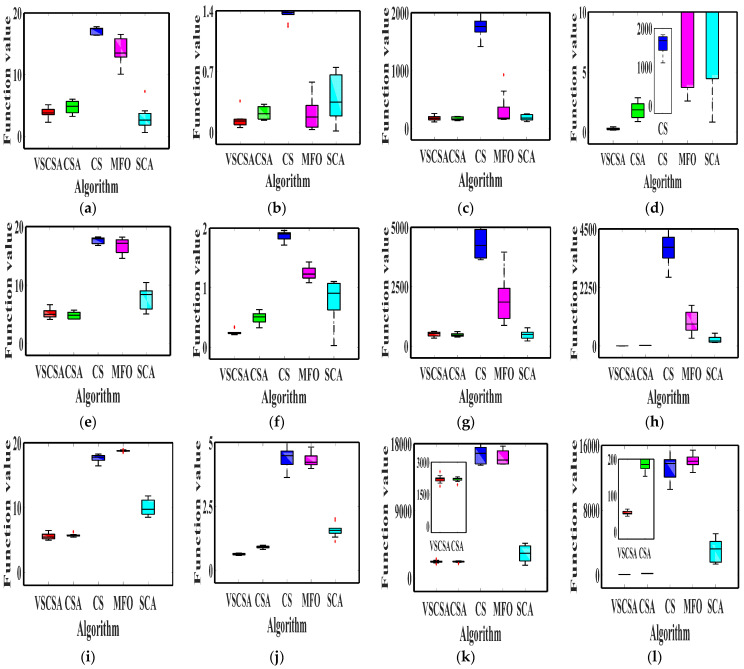
Box plot charts of high−dimension functions. (**a**) *f*_11(*D*=30)_; (**b**) *f*_12(*D*=30)_; (**c**) *f*_13(*D*=30)_; (**d**) *f*_14(*D*=30)_; (**e**) *f*_11(*D*=60)_; (**f**) *f*_12(*D*=60)_; (**g**) *f*_13(*D*=60)_; (**h**) *f*_14(*D*=60)_; (**i**) *f*_11(*D*=200)_; (**j**) *f*_12(*D*=200)_; (**k**) *f*_13(*D*=200)_; (**l**) *f*_14(*D=200*)_.

The box plot connects the two quartiles and connects the upper and lower edges to draw the box plot, and the median is in the middle of the box plot. If the box plot is narrower, the data is more concentrated. This paper gave algorithm box plots, as shown in Figure 4 and Figure 5. Compared with different algorithms in low-dimension box plots, VSCSA has the narrowest box plot except for *f*_8_, *f*_13_. In *f*_8_ and *f*_13_, CSA has the narrowest box plot. Compared with different algorithms in high-dimension box plots, VSCSA has the narrowest box plot except for *f*_11(*D*=60)_, *f*_13(*D*=60)_, *f*_11(*D*=200)_, *f*_13(*D*=200)_. In *f*_11(*D*=60)_, *f*_13(*D*=60)_, *f*_11(*D*=200)_, *f*_13(*D*=200)_, CSA has the narrowest box plot. Compared to the standard CSA algorithm, the VSCSA algorithm not only has a higher solving accuracy but also runs faster in most testing functions, which fully demonstrates that the VSCSA retains outstanding local search ability and is a significant improvement in global searching performances.

### 4.5. Sub-Sequence Runs Results

**Figure 6:**
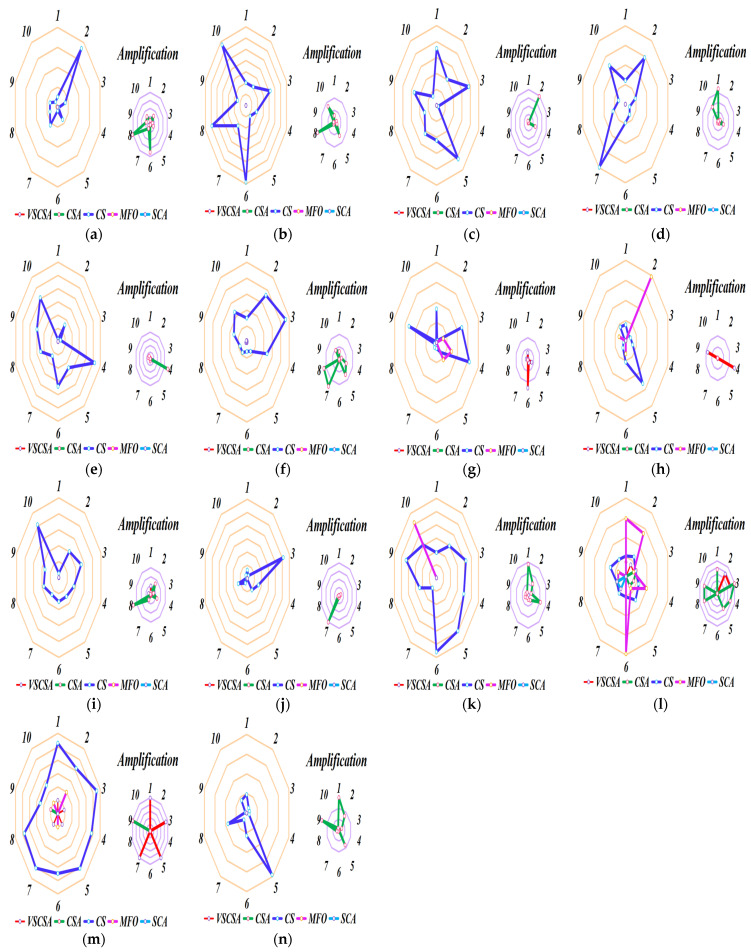
Sub-sequence runs radar charts of two−dimension functions. (**a**) *f*_1_; (**b**) *f*_2_; (**c**) *f*_3_; (**d**) *f*_4_; (**e**) *f*_5_; (**f**) *f*_6_; (**g**) *f*_7_; (**h**) *f*_8_; (**i**) *f*_9_; (**j**) *f*_10_; (**k**) *f*_11(*D*=2)_; (**l**) *f*_12(*D*=2)_; (**m**) *f*_13(*D*=2)_; (**n**) *f*_14(*D*=2)_.

**Figure 7:**
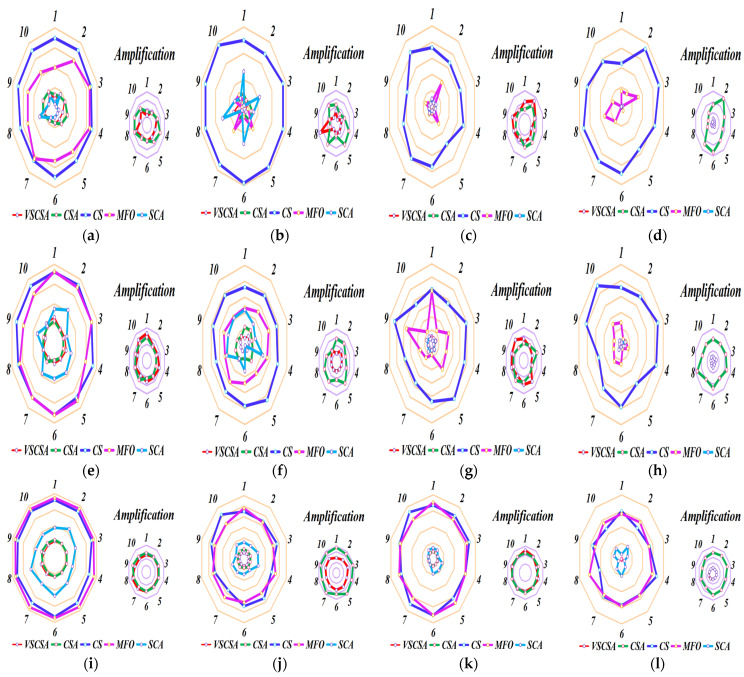
Sub-sequence runs radar charts of high−dimension functions. (**a**) *f*_11(*D*=30)_; (**b**) *f*_12(*D*=30)_; (**c**) *f*_13(*D*=30)_; (**d**) *f*_14(*D*=30)_; (**e**) *f*_11(*D*=60)_; (**f**) *f*_12(*D*=60)_; (**g**) *f*_13(*D*=60)_; (**h**) *f*_14(*D*=60)_; (**i**) *f*_11(*D*=200)_; (**j**) *f*_12(*D*=200)_; (**k**) *f*_13(*D*=200)_; (**l**) *f*_14(*D=200*)_.

Different axes are projected at equal angular intervals from the same center point, each axis represents a quantitative variable, and points on each axis are sequentially connected into lines or geometric shapes. Each variable has its axis, with equal distances between them, and all axes have the same scale. It is equivalent to a parallel coordinate map, which is arranged radially along the axis. This paper shows the basic statistical assessment obtained in sub-sequence runs of different algorithms. Ten sub-sequence runs are shown in Figure 6 and Figure 7. If the total length of polygon edges with different colors is longer, the lower the accuracy of the algorithm subsequence operations. For two-dimension amplification radar charts, CS subsequences have the largest radar charts except for *f*_12(*D*=2)_. MFO radar charts are larger than CSA radar charts in *f*_12(*D*=2)_. For high-dimension amplification radar charts, CS subsequences have the largest radar charts except for *f*_11(*D*=60)_, *f*_11(*D*=200)_ to *f*_14(*D*=200)_.

### 4.6. Search Path Results

**Figure 8:**
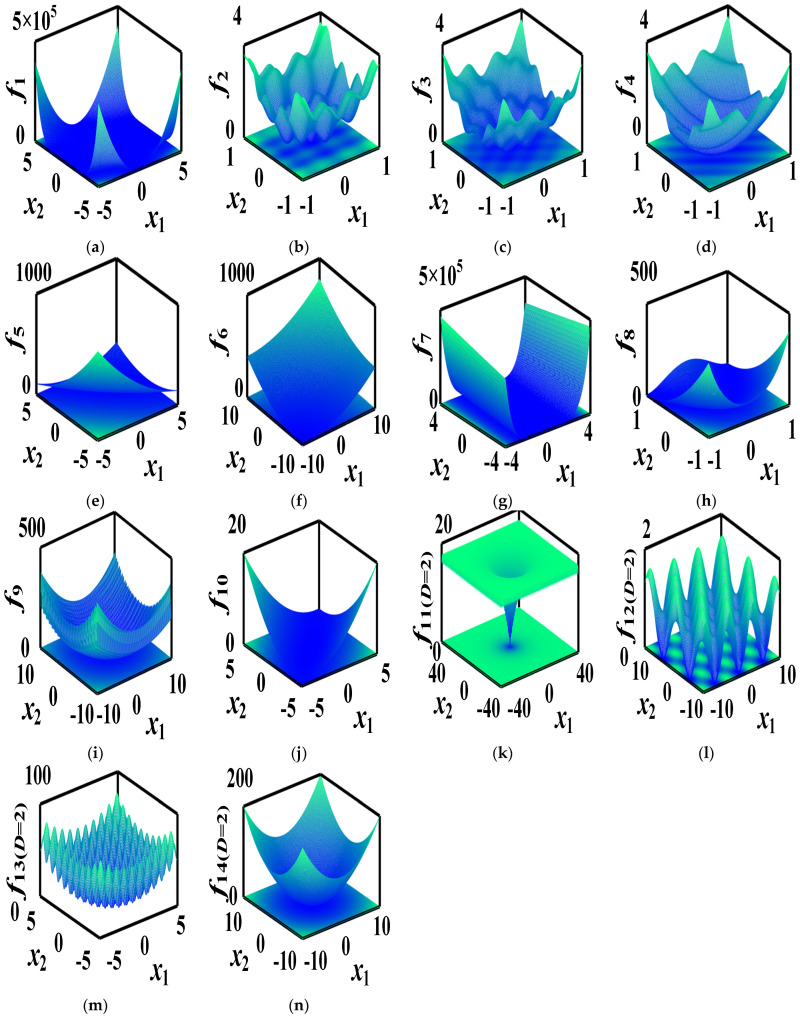
Three−dimension images of two-dimension functions. (**a**) *f*_1_; (**b**) *f*_2_; (**c**) *f*_3_; (**d**) *f*_4_; (**e**) *f*_5_; (**f**) *f*_6_; (**g**) *f*_7_; (**h**) *f*_8_; (**i**) *f*_9_; (**j**) *f*_10_; (**k**) *f*_11(*D*=2)_; (**l**) *f*_12(*D*=2)_; (**m**) *f*_13(*D*=2)_; (**n**) *f*_14(*D*=2)_.

**Figure 9:**
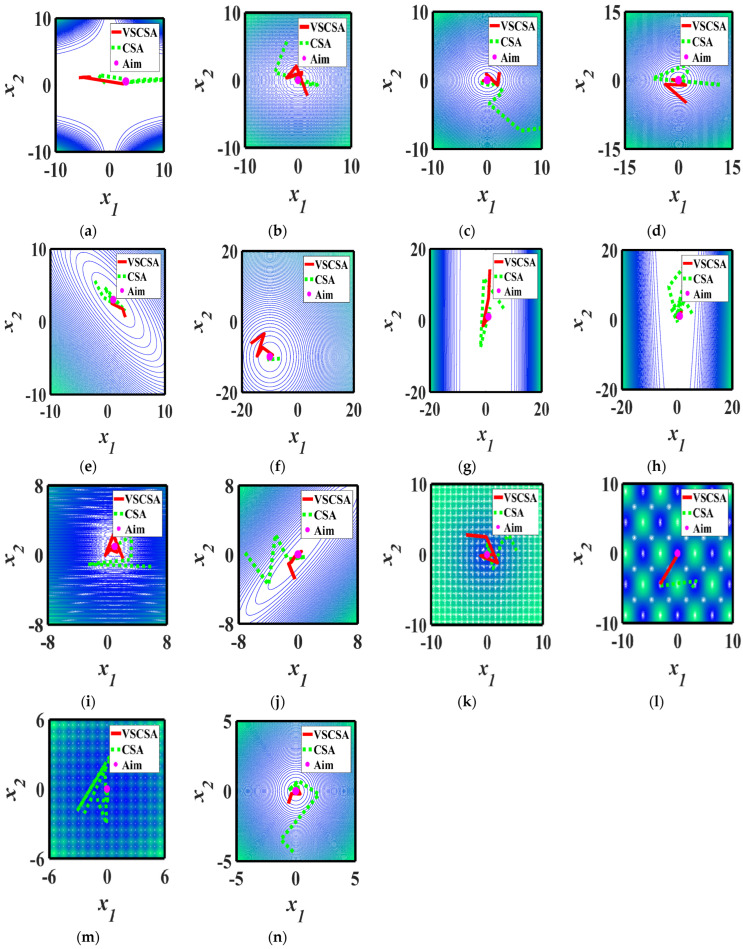
Search paths. (**a**) *f*_1_; (**b**) *f*_2_; (**c**) *f*_3_; (**d**) *f*_4_; (**e**) *f*_5_; (**f**) *f*_6_; (**g**) *f*_7_; (**h**) *f*_8_; (**i**) *f*_9_; (**j**) *f*_10_; (**k**) *f*_11(*D*=2)_; (**l**) *f*_12(*D*=2)_; (**m**) *f*_13(*D*=2)_; (**n**) *f*_14(*D*=2)_.

To test the structural reliability analysis, the computational efficiency, and the accuracy of the proposed algorithm, three-dimensional images of two-dimension functions are given in Figure 8, while the VSCSA path and the CSA path are refracted to a two-dimension plane in Figure 9. The red straight line is the VSCSA searching path, the green dashed line is the CSA searching path, and the pink dot is the theoretical optimal position. CSA searching paths have many short repeat searching paths and occasional long searching paths. The VSCSA algorithm has a strong performance in population diversity, representing the global optimal performance. In the early stage of the algorithm searching process, the VSCSA can quickly traverse and explore the entire solution region, lock in the approximate range of the global optimal solution, and ensure the diversity of the population. At the end stage of the algorithm searching process, the reduction of differences between individuals makes the searching process jump out of local vortices and find the ideal optimization solution, which can improve the algorithm global convergence ability.

### 4.7. Wilcoxon Rank Sum Test Results

In the process of detecting algorithms, more different experimental results often appear. When comparing and analyzing algorithms, conclusions cannot be drawn solely based on differences in a few results, so statistical analysis should be conducted to test the significance of differences in the data. The Wilcoxon rank sum test result is the *p*-value. If the *p*-value is greater than 0.05, there is no significant change for two sets of data. If the *p*-value is less than 0.05, two algorithm performances are significant. In Table 4, N means that the computer cannot give the *p*-value because of the too-large or too-small *p*-value. In function *f*_8_, *f*_13(*D*=2)_, *f*_11(*D*=30_), *f*_13(*D*=30)_, *f*_11(*D*=60)_, *f*_13(*D*=60)_, *f*_11(*D*=200)_, *f*_13(*D*=200)_, the *p*-value in CSA is larger than 0.05. In function *f*_4_, *f*_10_, *f*_11(*D*=2)_, *f*_13(*D*=2)_, *f*_12(*D*=30)_, *f*_13(*D*=30)_, the *p*-value in MFO is larger than 0.05. In function *f*_11(*D*=2)_, *f*_12(*D*=2)_, *f*_11(*D*=30)_, *f*_13(*D*=30)_, *f*_13(*D*=60)_, the *p*-value in SCA is larger than 0.05. For other algorithms, the Wilcoxon rank sum test results are all less than 0.05. From the results of the Wilcoxon rank sum test by VSCSA, the searching accuracy of the algorithm has been significantly improved, and the improved algorithm is significantly better than the standard CSA in terms of searching accuracy and speed.

### 4.8. Algorithm Ranking Results

**Figure 10:**
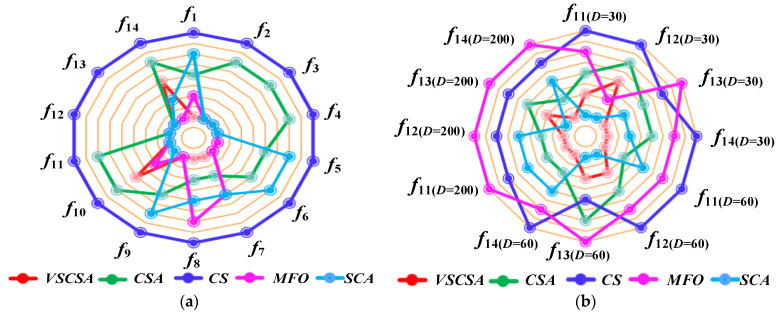
Algorithm ranking figures. (**a**) Two-dimension functions. (**b**) High-dimension functions.

Algorithm ranking radar charts are shown in Figure 10. The positions of different colored dots in the radar image represent the algorithm searching accuracy. If the algorithm point is close to the center point, the algorithm has a high ranking. It can be seen that the VSCSA surrounds the center point. From the radar graph, it can be seen that VSCSA has the best results among multiple test functions and has the highest searching accuracy among comparison algorithms. Although VSCSA did not achieve comprehensive advantages in some test functions, it achieved optimal searching results in more than half of the test functions, indicating that VSCSA has strong competitiveness. It can be seen that the proposed VSCSA in this paper greatly enhances the CSA searching performance.

## 5. CEC2017 Test Function Experiment Results

### 5.1. Experiment Environments

The IEEE Congress on Evolutionary Computation (CEC) is one of the largest and most significant conferences within Evolutionary Computation (EC). CEC test functions under the CEC conference series are among the widely used benchmarks to test different algorithms. CEC2017 is the test function in the 2017 CEC conference. CEC2017 consists of different problems, including Unimodal, Multimodal, Hybrid, and Composition functions. To further show the proposed algorithm, this paper selected CEC2017 in *F*_1_ to *F*_20_. *F*_1_ to *F*_20_ of CEC2017 are given in Table 5. In Table 5, *D* is the searching dimension, *F*_min_ is the idea function value, and range is the searching scope. *F*_1_ and *F*_2_ are Unimodal Functions, *F*_3_ to *F*_9_ are Simple Multimodal Functions, *F*_10_ to *F*_19_ are Hybrid Functions, and *F*_20_ is the Composition. In this paper, the proposed method compares with state-of-the-art algorithms (SOTA) in recent years. SOTA includes the bald eagle search algorithm (BES) [48], COOT bird algorithm (COOT) [49], wild horse optimizer (WHO) [18], and whale optimization algorithm (WOA) [10]. All algorithm parameters were selected according to original literature. The population size and the maximum number of iterations were 20 and 5000, respectively. To obtain a fair comparison result, all algorithms independently ran 10 times in MATLAB(R2016b). The experimental environment was the Windows 7 operating system, Intel (R) Core (TM) i3-7100CPU, 8GBRAM.

### 5.2. Experiment Results

The statistical results of algorithms on CEC2017 benchmark functions are shown in Table 6. In Table 6, Min, Max, and Var mean the minimum value, the maximum value, and the variance deviation. For Unimodal Functions, VSCSA, CSA, BES, COOT, and WHO can obtain the ideal value in *F*_1_. All six algorithms can obtain the ideal value in *F*_2_. For Simple Multimodal Functions, all six algorithms can obtain the ideal value in *F*_3_ to *F*_9_. For Hybrid Functions, all six algorithms can obtain the ideal value in *F*_10_ and *F*_11_. CSA can obtain the minimum value in *F*_12_. BES can obtain the minimum value in *F*_17_. COOT can obtain the minimum value in *F*_14_ *F*_16_. WHO can obtain the minimum value in *F*_13_ *F*_15_ *F*_18_ *F*_19_. For the Composition, COOT can obtain the minimum value in *F*_20_.

**Figure 11:**
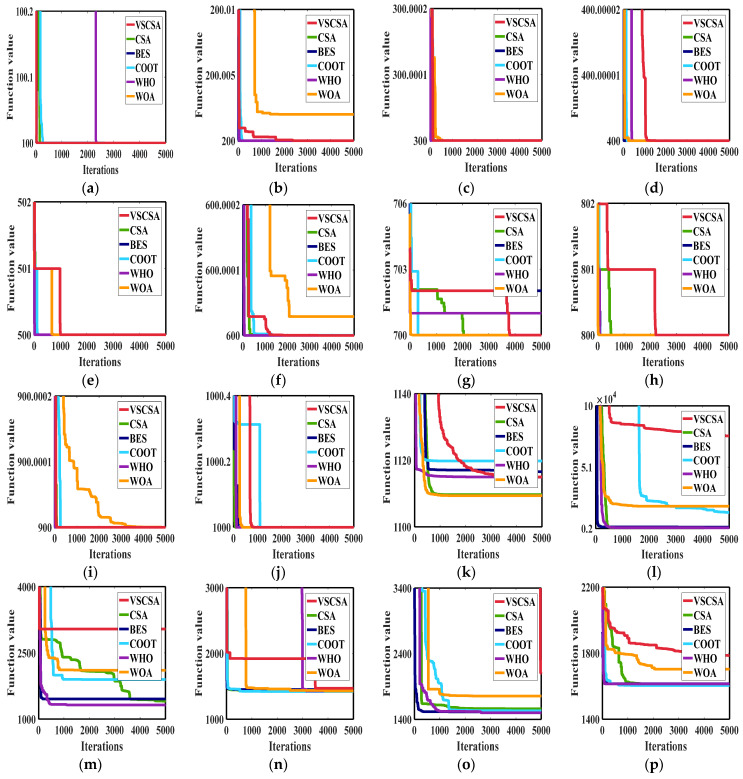

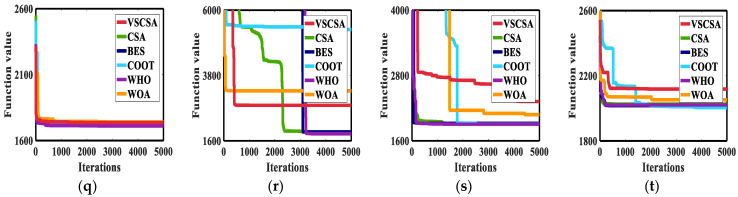
Iteration curves of CEC2017 functions. (**a**) *F*_1_; (**b**) *F*_2_; (**c**) *F*_3_; (**d**) *F*_4_; (**e**) *F*_5_; (**f**) *F*_6_; (**g**) *F*_7_; (**h**) *F*_8_; (**i**) *F*_9_; (**j**) *F*_10_; (**k**) *F*_11_; (**l**) *F*_12_; (**m**) *F*_13_; (**n**) *F*_14_; (**o**) *F*_15_; (**p**) *F*_16_; (**q**) *F*_17_; (**r**) *F*_18_; (**s**) *F*_19_; (**t**) *F*_20_.

Figure 11 gives the best iteration curves of different algorithms in 10 independent runs. From Figure 11 we can see that VSCSA has the fastest initial iteration speed in function *F*_1_, *F*_3_, and *F*_9_. And VSCSA has the fastest iteration speed in the later stage for function *F*_2_, *F*_4_ to *F*_8_, and *F*_10_. VSCSA has the slowest iteration speed in *F*_12_, *F*_15_, and *F*_16_.

**Figure 12:**
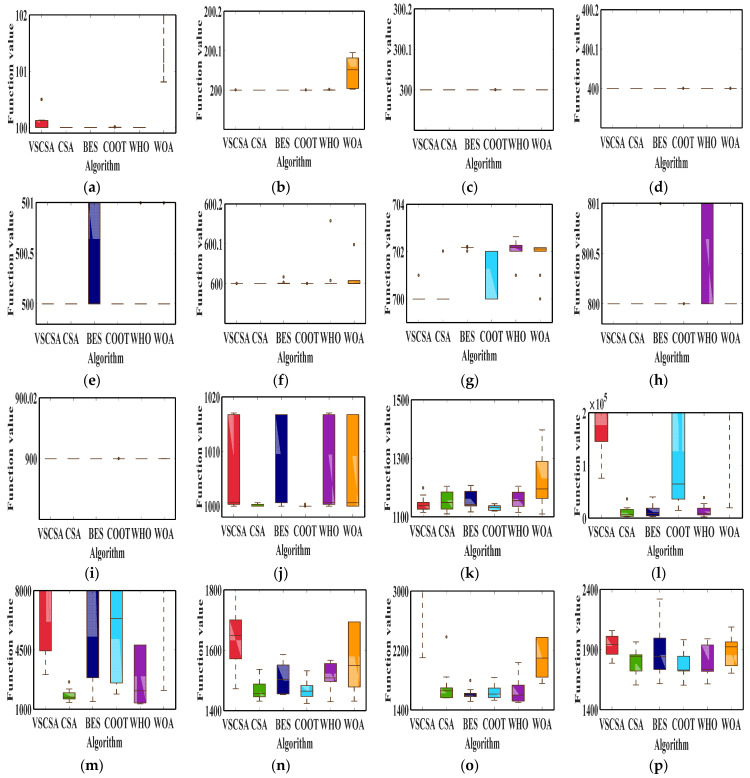

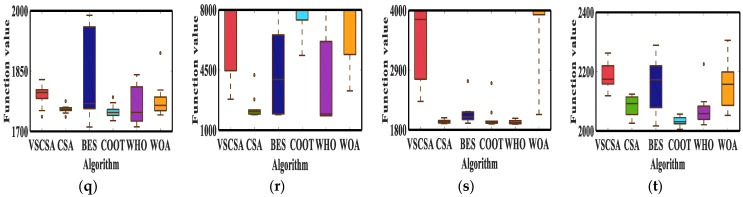
Box plot charts of CEC2017 functions. (**a**) *F*_1_; (**b**) *F*_2_; (**c**) *F*_3_; (**d**) *F*_4_; (**e**) *F*_5_; (**f**) *F*_6_; (**g**) *F*_7_; (**h**) *F*_8_; (**i**) *F*_9_; (**j**) *F*_10_; (**k**) *F*_11_; (**l**) *F*_12_; (**m**) *F*_13_; (**n**) *F*_14_; (**o**) *F*_15_; (**p**) *F*_16_; (**q**) *F*_17_; (**r**) *F*_18_; (**s**) *F*_19_; (**t**) *F*_20_.

Figure 12 gives box plots for different algorithms after 10 independent runs. VSCSA has the narrowest box plot in function *F*_2_ to *F*_9_. CSA has the narrowest box plot in function *F*_12_, *F*_13,_ and *F*_18_. BES has the narrowest box plot in function *F*_15_. COOT has the narrowest box plot in function *F*_10_, *F*_11_, *F*_14_, *F*_16_, *F*_17_, *F*_19_, and *F*_20_. VSCSA has the worst box plot in function *F*_1_ and *F*_15_. BES has the worst box plot in function *F*_5_, *F*_16_, *F*_17_, and *F*_20_. COOT has the worst box plot in function *F*_7_. WHO has the worst box plot in function *F*_8_. WOA has the worst box plot in function *F*_6_, *F*_11_, *F*_12_, *F*_13_, *F*_14_, *F*_18_, and *F*_19_. For *F*_10_, VSCSA, BES, WHO, and WOA have large box plots.

**Figure 13:**
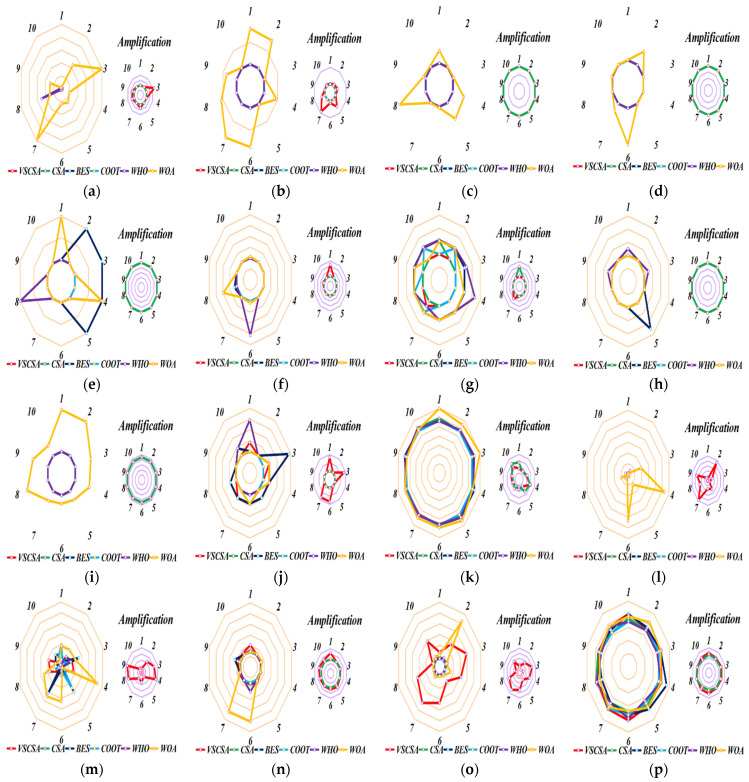

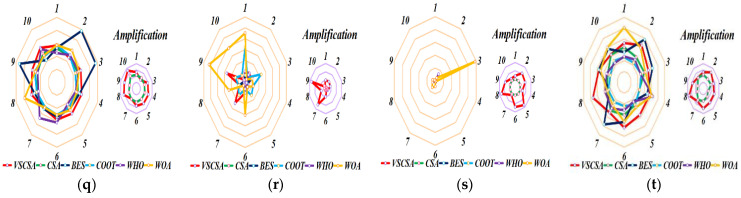
Sub-sequence runs radar charts of CEC2017 functions. (**a**) *F*_1_; (**b**) *F*_2_; (**c**) *F*_3_; (**d**) *F*_4_; (**e**) *F*_5_; (**f**) *F*_6_; (**g**) *F*_7_; (**h**) *F*_8_; (**i**) *F*_9_; (**j**) *F*_10_; (**k**) *F*_11_; (**l**) *F*_12_; (**m**) *F*_13_; (**n**) *F*_14_; (**o**) *F*_15_; (**p**) *F*_16_; (**q**) *F*_17_; (**r**) *F*_18_; (**s**) *F*_19_; (**t**) *F*_20_.

Figure 13 gives radar charts for different algorithms after 10 independent runs. For Figure 13, VSCSA subsequences have the largest radar charts for function *F*_15_. BES has the largest radar charts for function *F*_5_ and *F*_8_. WHO has the largest radar charts for function *F*_6_. WOA has the largest radar charts for function *F*_1_ to *F*_4_, *F*_9_, *F*_12_, *F*_14_, *F*_18,_ and *F*_19_. For function *F*_7_, *F*_10_, *F*_11_, *F*_13_, *F*_16_, *F*_17,_ and *F*_20_, many algorithms have large radar charts.

Table 7 shows the Wilcoxon rank sum test results. In Table 7, N means that the computer cannot give the *p*-value because of the too-large or too-small *p*-value. In function *F*_7_, *F*_11_, the *p*-value in CSA is larger than 0.05. In functions *F*_6_, *F*_8_, *F*_10_, *F*_11_, *F*_13_, *F*_16_, *F*_17_, *F*_20_, the *p*-value in BES is larger than 0.05. In function *F*_1_, *F*_3_, *F*_6_, *F*_8_, *F*_9_, *F*_11_, *F*_13_, *F*_18_, the *p*-value in COOT is larger than 0.05. In function *F*_2_, *F*_5_, *F*_6_, *F*_8_, *F*_10_, *F*_11_, *F*_14_, *F*_17_, the *p*-value in WHO is larger than 0.05. In function *F*_10_, *F*_12_, *F*_10_, *F*_14_, *F*_16_ to *F*_20_, the *p*-value in WOA is larger than 0.05. For other algorithms, the Wilcoxon rank sum test results are all less than 0.05.

**Table 5:** Basic information of CEC2017 benchmark functions.

No.	Function	*D*	Range	*F*_min_
*F*_1_	Shifted and Rotated Bent Cigar Function	2	[−100, 100]	100
*F*_2_	Shifted and Rotated Zakharov Function	2	[−100, 100]	200
*F*_3_	Shifted and Rotated Rosenbrock’s Function	2	[−100, 100]	300
*F*_4_	Shifted and Rotated Rastrigin’s Function	2	[−100, 100]	400
*F*_5_	Shifted and Rotated Expanded Scaffer’s F6 Function	2	[−100, 100]	500
*F*_6_	Shifted and Rotated Lunacek Bi-Rastrigin Function	2	[−100, 100]	600
*F*_7_	Shifted and Rotated Non-Continuous Rastrigin’s Function	2	[−100, 100]	700
*F*_8_	Shifted and Rotated Levy Function	2	[−100, 100]	800
*F*_9_	Shifted and Rotated Schwefel’s Function	2	[−100, 100]	900
*F*_10_	Hybrid Function 1 (N = 3)	2	[−100, 100]	1000
*F*_11_	Hybrid Function 2 (N = 3)	10	[−100, 100]	1100
*F*_12_	Hybrid Function 3 (N = 3)	10	[−100, 100]	1200
*F*_13_	Hybrid Function 4 (N = 4)	10	[−100, 100]	1300
*F*_14_	Hybrid Function 5 (N = 4)	10	[−100, 100]	1400
*F*_15_	Hybrid Function 6 (N = 4)	10	[−100, 100]	1500
*F*_16_	Hybrid Function 6 (N = 5)	10	[−100, 100]	1600
*F*_17_	Hybrid Function 6 (N = 5)	10	[−100, 100]	1700
*F*_18_	Hybrid Function 6 (N = 5)	10	[−100, 100]	1800
*F*_19_	Hybrid Function 6 (N = 6)	10	[−100, 100]	1900
*F*_20_	Composition Function 1 (N = 3)	10	[−100, 100]	2000

**Table 6:** Comparison of results for CEC2017 benchmark functions.

Function	Metric	VSCSA	CSA	BES	COOT	WHO	WOA
*F*_1_	Min	100.0000	100.0000	100.0000	100.0000	100.0000	100.8089
	Max	100.4967	100.0000	100.0000	100.0079	2476.9326	4991.5872
	Var	0.0239	0	0	5.8721 × 10^−6^	5.6498 × 10^5^	3.0578 × 10^6^
*F*_2_	Min	200.0000	200.0000	200.0000	200.0000	200.0000	200.0020
	Max	200.0000	200.0000	200.0000	200.0000	200.0012	200.0951
	Var	3.2226 × 10^−13^	0	0	7.7463 × 10^−11^	1.6905 × 10^−7^	1.3605 × 10^−3^
*F*_3_	Min	300.0000	300.0000	300.0000	300.0000	300.0000	300.0000
	Max	300.0000	300.0000	300.0000	300.0000	300.0000	300.0000
	Var	0	0	0	3.5902 × 10^−28^	0	9.2862 × 10^−22^
*F*_4_	Min	400.0000	400.0000	400.0000	400.0000	400.0000	400.0000
	Max	400.0000	400.0000	400.0000	400.0000	400.0000	400.0000
	Var	0	0	0	1.3381 × 10^−21^	6.9650 × 10^−26^	2.1903 × 10^−14^
*F*_5_	Min	500.0000	500.0000	500.0000	500.0000	500.0000	500.0000
	Max	500.0000	500.0000	500.9950	500.0000	500.9950	500.9950
	Var	0	0	0.2640	0	0.1760	0.1760
*F*_6_	Min	600.0000	600.0000	600.0000	600.0000	600.0000	600.0000
	Max	600.0002	600.0000	600.0164	600.0000	600.1573	600.0976
	Var	5.1834 × 10^−9^	0	2.6128 × 10^−5^	8.0042 × 10^−11^	0.0025	0.0009
*F*_7_	Min	700.0000	700.0000	702.0163	700.0000	700.9950	700.0000
	Max	700.9950	702.0163	702.2136	702.0163	704.7119	702.1708
	Var	0.0990	0.4066	0.0027	1.0842	0.8721	0.5292
*F*_8_	Min	800.0000	800.0000	800.0000	800.0000	800.0000	800.0000
	Max	800.0000	800.0000	804.9748	800.0000	800.9950	800.0000
	Var	0	0	2.4639	5.7443 × 10^−27^	0.2310	4.8755 × 10^−24^
*F*_9_	Min	900.0000	900.0000	900.0000	900.0000	900.0000	900.0000
	Max	900.0000	900.0000	900.0000	900.0000	900.0000	900.0000
	Var	0	0	0	5.7443 × 10^−27^	0	8.2264 × 10^−14^
*F*_10_	Min	1000.0000	1000.0000	1000.0000	1000.0000	1000.0000	1000.0000
	Max	1017.0694	1000.6243	1074.9496	1000.3122	1058.5045	1016.7572
	Var	71.9334	0.0444	465.5578	0.0097	337.6272	63.1675
*F*_11_	Min	1114.9624	1109.6715	1116.5732	1119.8084	1114.9368	1109.3504
	Max	1198.3403	1204.5114	1207.1855	1144.6353	1204.4832	1396.9177
	Var	663.0311	1057.8398	958.1988	70.7817	889.1115	1.0239 × 10^4^
*F*_12_	Min	7.5856 × 10^4^	2604.2573	3128.5674	1.4793 × 10^4^	2.5114 × 10^3^	1.9748 × 10^4^
	Max	9.7061 × 10^5^	3.6182 × 10^4^	4.0349 × 10^4^	5.7144 × 10^5^	3.8765 × 10^4^	1.0693 × 10^7^
	Var	1.0431 × 10^+11^	1.1384 × 10^8^	1.4756 × 10^8^	4.7970 × 10^+10^	1.3494 × 10^8^	1.48761 × 10^+13^
*F*_13_	Min	3041.5027	1403.7263	1455.2229	1895.3341	1318.7813	2105.0934
	Max	1.8156 × 10^4^	2602.3091	3.1311 × 10^4^	2.5062 × 10^4^	1.3722 × 10^4^	5.2478 × 10^4^
	Var	3.8814 × 10^7^	1.2894 × 10^5^	1.3522 × 10^8^	6.6879 × 10^7^	2.0050 × 10^7^	2.3650 × 10^8^
*F*_14_	Min	1472.2338	1431.3035	1453.1541	1423.2821	1429.3626	1431.4869
	Max	2069.1500	1536.7192	2282.9765	1531.6019	2297.9909	5143.6059
	Var	3.1788 × 10^4^	1113.3125	6.3312 × 10^4^	1214.3278	6.3780 × 10^4^	2.2832 × 10^6^
*F*_15_	Min	2102.0812	1561.6616	1514.5328	1530.4193	1501.4497	1755.3194
	Max	8248.5418	2380.6647	1.7944 × 10^3^	1835.0032	2035.3400	1.0501 × 10^4^
	Var	4.5125 × 10^6^	6.0137 × 10^4^	6405.4267	9095.8887	2.7671 × 10^4^	7.1257 × 10^6^
*F*_16_	Min	1785.9923	1605.1444	1614.9232	1604.0291	1614.1054	1703.0662
	Max	2058.8581	1962.8200	2319.6410	1980.6734	1989.2964	2086.8136
	Var	9501.8979	1.0527 × 10^4^	3.8956 × 10^4^	1.0911 × 10^4^	1.9357 × 10^4^	1.4474 × 10^4^
*F*_17_	Min	1736.2506	1735.7399	1711.0671	1726.6221	1711.3399	1741.0307
	Max	1828.8309	1774.8088	1988.8544	1784.6624	1841.2146	1894.6790
	Var	756.9156	102.0301	1.1332 × 10^4^	297.7841	2068.8956	2046.5244
*F*_18_	Min	2795.7978	1899.0415	1900.8064	5348.1482	1837.5630	3284.2186
	Max	2.6628 × 10^4^	4194.3397	9071.9493	3.2147 × 10^4^	8313.3384	5.2491 × 10^4^
	Var	6.6663 × 10^7^	4.9868 × 10^5^	7.3939 × 10^6^	6.8506 × 10^7^	6.0211 × 10^6^	3.3418 × 10^8^
*F*_19_	Min	2323.2878	1914.0998	1919.9589	1906.5796	1905.3476	2080.4660
	Max	5351.9820	2022.0524	2.6936 × 10^3^	2656.9216	3.2933 × 10^4^	2.4271 × 10^5^
	Var	1.2781 × 10^6^	1145.4605	4.8170 × 10^4^	5.2873 × 10^4^	9.6078 × 10^7^	5.3813 × 10^9^
*F*_20_	Min	2118.6468	2025.8065	2016.9142	2004.9954	2020.8626	2052.4900
	Max	2262.7814	2123.8647	2289.2135	2056.6773	2224.7862	2305.7172
	Var	1758.7689	1230.6049	9242.5172	231.5231	3534.4072	5965.3790

**Table 7:** Comparison of the Wilcoxon rank sum test results in CEC2017 functions.

Function	CSA	BES	COOT	WHO	WOA
*F*_1_	0.00075	0.00075	0.51989	0.02384	0.00018
*F*_2_	0.00006	0.00006	0.03764	0.47100	0.00018
*F*_3_	N	N	0.36812	N	0.00006
*F*_4_	N	N	0.03498	0.01493	0.00006
*F*_5_	N	0.03359	N	0.16749	0.00023
*F*_6_	0.01493	0.57148	0.10957	0.39943	0.00069
*F*_7_	1.00000	0.00007	0.04981	0.00010	0.00012
*F*_8_	N	0.16808	0.36812	0.07672	0.00023
*F*_9_	N	N	0.36812	N	0.00006
*F*_10_	0.00978	0.35909	0.00445	1.00000	0.96975
*F*_11_	0.52052	0.42736	0.34470	0.27304	0.01726
*F*_12_	0.00018	0.00018	0.04515	0.00018	0.42736
*F*_13_	0.00018	0.96985	0.42736	0.01402	0.03764
*F*_14_	0.00101	0.03121	0.00077	0.06402	0.30749
*F*_15_	0.00025	0.00018	0.00018	0.00018	0.00911
*F*_16_	0.00911	0.27304	0.00361	0.01726	0.47268
*F*_17_	0.01402	0.57075	0.00459	0.18588	0.14047
*F*_18_	0.00033	0.02113	0.27304	0.00283	0.12122
*F*_19_	0.00018	0.00033	0.00033	0.00283	0.05390
*F*_20_	0.00033	0.73373	0.00018	0.00220	0.34470

## 6. Engineering Applications

### 6.1. Three Bar Truss Design Problem

**Figure 14:**
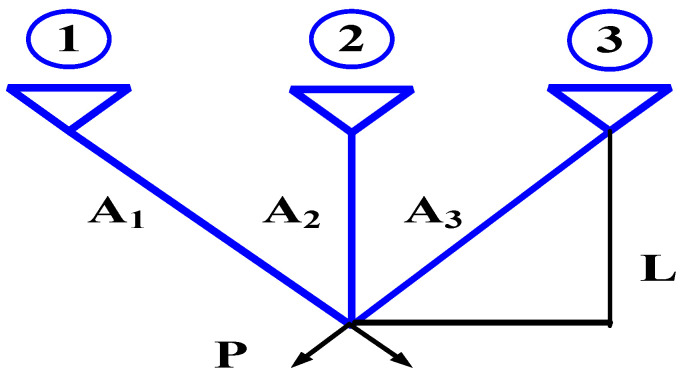
Three bar truss design problem.

The three bar truss design problem is a civil engineering problem, and the weight of the bar structure is the key problem in the Gear Train Problem which owns a problematic and constrained space. The constraints of this problem are based on the stress constraints of each bar. Figure 14 is the structural diagram of the three bar truss design problem. A_1_ A_2_ A_3_ respectively represents the length of the bar, P means the force value, L means the space length. 

This problem can be described mathematically as follows:

$$\begin{array}{l} Consider\;\;\;\;\;\;\vec{X}=\left[x_{1}x_{2}\right]=\left[A_{1}A_{1}\right]\\ Minimize\;\;\;\;\;f\left(\vec{X}\right)=\left(2\sqrt{2}x_{1}+x_{2}\right)\times L\\ Subject\;\;to\;\;\;\;g_{1}\left(\vec{X}\right)=\frac{2\sqrt{2}x_{1}+x_{2}}{\sqrt{2}x_{1}^{2}+2x_{1}x_{2}}\times P-\sigma \leq 0\\ \;\;\;\;\;\;\;\;\;\;\;\;\;\;\;\;\;\;\;\;g_{2}\left(\vec{X}\right)=\frac{x_{2}}{\sqrt{2}x_{1}^{2}+2x_{1}x_{2}}\times P-\sigma \leq 0\\ \;\;\;\;\;\;\;\;\;\;\;\;\;\;\;\;\;\;\;\;\;g_{3}\left(\vec{X}\right)=\frac{1}{\sqrt{2}x_{2}+x_{1}}\times P-\sigma \leq 0\\ \;\;\;0\leq x_{1},x_{2}\leq 1\begin{array}{cc} & P=2\mathrm{KN}/{\mathrm{cm}}^{2}\begin{array}{cc} & L=100\mathrm{cm}\begin{array}{cc} & \sigma =2\mathrm{KN}/{\mathrm{cm}}^{2} \end{array} \end{array} \end{array} \end{array}$$

In this paper, basic CSA were selected for the CSA literature. Comparison algorithms and parameters selected the algorithm literature [50], with each method tested 30 times with 1000 iterations and a maximum of 60,000 number function evaluations (NFEs). The results of best, mean, minimum values, maximum values, and the standard deviation value are given in Table 8.

The VSCSA Min value is the same as the CSA Min value and the WHO Min value. The VSCSA Max value is larger than the CSA Max value. WHO and CSA obtain the less Max value and the Avg value. WHO obtains the less Std value. AEFA obtains the worst Min value, the Max value, the Std value, and the Avg value.

### 6.2. The Gear Train Problem

**Figure 15:**
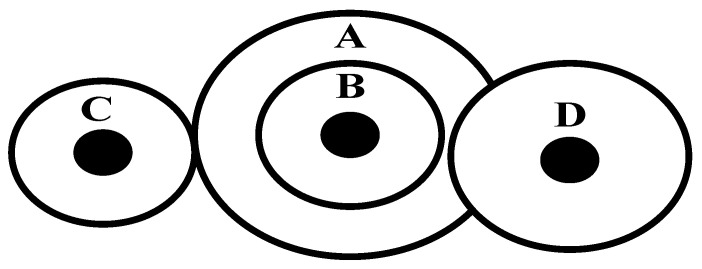
Gear train problem.

The cost of the gear ratio of the gear train is the key problem in the Gear Train Problem which owns only four parameters in boundary constraints. Four parameters are discrete because each gear should have an integral number of teeth in this problem. Discrete variables add different complexities for this problem. Figure 15 is the structural diagram of the Gear Train Problem. Four parameters are the numbers of teeth on the gears: nA, nB, nC, and nD. A, B, C, and D mean centre points.

This problem can be described mathematically as follows:

$$\begin{array}{l} Consider\;\;\;\;\;\;\;\vec{X}=\left[x_{1}x_{2}x_{3}x_{4}\right]\\ Minimize\;\;\;\;\;\;\;f\left(\vec{X}\right)={\left(\frac{1}{6.931}-\frac{x_{3}x_{2}}{x_{1}x_{4}}\right)}^{2}\\ Subject\;\;to\;\;\;\;12\leq x_{1},x_{2},x_{3},x_{4}\leq 60 \end{array}$$

In this paper, basic CSA was selected for the CSA literature. Comparison algorithms and parameters selected the algorithm literature, the number of population sizes is set to 50, and the maximum number of iterations is set to 1000. All algorithms are executed for 30 independent runs. The results of the best, mean, minimum values, maximum values, and the standard deviation value are given in Table 9. Comparison algorithms include CS [46], FPA [50], FSA [51], SA [52], and SCA [14]. The VSCSA Min value is the same as the CSA Min value. SCA obtains the worst Min value. SCA obtains the worst Max value, Std value, and Avg value. The VSCSA Min value, Max value, Std value, and Avg value are larger than those of CSA. There is no specific algorithm that can perfectly solve all engineering problems. Different algorithms can be selected for different engineering problems.

## 7. Conclusions

In this paper, VSCSA is introduced to solve function problems. The proposed algorithm uses the cosine function to enhance the CSA searching ability. VSCSA has strong problem applicability and can effectively find the global optimum in a short iteration period, greatly improving the solution accuracy. In conclusion, the proposed algorithm VSCSA has significant advantages over CSA in CEC-2017 fitness values, iteration curves, box plots, and search paths. In addition, the Wilcoxon test results statistically indicate differences between VSCSA and other comparative algorithms. Engineering applications show that the proposed algorithm has strong competitiveness. The above data and conclusions indicate that the improvement strategy proposed in this paper has achieved good results, greatly improving the performances of the original CSA. Many algorithms have been applied to specific fields such as medicine, aerospace, and industry and have achieved good results. Therefore, combining VSCSA with practical problems in specific fields is a direction for future research.

## Figures and Tables

**Table 1:** Basic information of benchmark functions.

Name	Function	*D*	Range	*f*_min_
Beale	*f*_1_(*x*) = (1.5 − $x_{1}$ + $x_{1}$$x_{2}$)^2^ + (2.25 − $x_{1}$ + $x_{1}$$x_{2}^{2}$)^2^ + (2.625 − $x_{1}$ + $x_{1}$$x_{2}^{3}$)^2^	2	[−50, 50]	0
Bohachevsky01	*f*_2_(*x*) = $x_{1}^{2}$ + 2$x_{2}^{2}$ − 0.3cos(3π$x_{1}$) − 0.4cos(4π$x_{2}$) + 0.7	2	[−50, 50]	0
Bohachevsky02	*f*_3_(*x*) = $x_{1}^{2}$+ 2$x_{2}^{2}$ − 0.3cos(3π$x_{1}$) cos(4π$x_{2}$) + 0.3	2	[−50, 50]	0
Bohachevsky03	*f*_4_(*x*) = $x_{1}^{2}$ + 2$x_{2}^{2}$ − 0.3cos(3π*x*_1_ + 4π$x_{2}$) + 0.3	2	[−50, 50]	0
Booth	*f*_5_(*x*) = ($x_{1}$ + 2*x*_2_ − 7)^2^ + (2*x*_1_ + *x*_2_ *−* 5)^2^	2	[−50, 50]	0
Brent	*f*_6_(*x*) = ($x_{1}$ + 10)^2^ + ($x_{2}$ + 10)^2^ + *e*^−$x_{1}^{2}$−$x_{2}^{2}$^	2	[−50, 50]	0
Cube	*f*_7_(*x*) = 100(*x*_2_ − $x_{1}^{3}$)^2^ + (1 − $x_{1}$)^2^	2	[−50, 50]	0
Leon	*f*_8_ (*x*) = 100(*x*_2_ − $x_{1}^{2}$)^2^ + (1 − $x_{1}$)^2^	2	[−50, 50]	0
Levy13	*f*_9_(*x*) = sin^2^(3π*x*_1_) + (*x*_1_ − 1)^2^[1 + sin^2^(3π*x*_2_)] + (*x*_2_ − 1)^2^[1 + sin^2^(2π*x*_2_)]	2	[−50, 50]	0
Matyas	*f*_10_(*x*) = 0.26($x_{1}^{2}$ + $x_{2}^{2}$) − 0.48 $x_{1}$$x_{2}$	2	[−50, 50]	0
Ackley 01	$\begin{array}{l} f_{11}\left(x\right)=-20\exp\left(-0.2\sqrt{\frac{1}{D}\sum_{i=1}^{D}x_{i}^{2}}\right)-\exp\left(\frac{1}{D}\sum_{i=1}^{D}\cos\left(2\pi x_{i}\right)\right)\\ +20+\exp(1) \end{array}$	2/30/60/200	[−20, 20]	0
Griewank	$f_{12}\left(x\right)=\sum_{i=1}^{D}\left(x_{i}^{2}/4000\right)-\prod_{i=1}^{D}\cos\left(x_{i}/\sqrt{i}\right)+1$	2/30/60/200	[−20, 20]	0
Rastrigin	$f_{13}\left(x\right)=10D+\sum_{i=1}^{D}\left(x_{i}^{2}-10\cos\left(2\pi x_{i}\right)\right)$	2/30/60/200	[−20, 20]	0
Sphere	$f_{14}\left(x\right)=\sum_{i=1}^{D}x_{i}^{2}$	2/30/60/200	[−20, 20]	0

**Table 2:** Comparison of results for two-dimension functions.

Function	Metric	VSCSA	CSA	CS	MFO	SCA
*f*_1_	Min	1.2634 × 10^−31^	1.1569 × 10^−16^	0.0436	8.2737 × 10^−22^	0.0001
	Max	6.4206 × 10^−16^	8.2586 × 10^−15^	1.8208	0.0358	0.0035
	Ave	7.9525 × 10^−17^	2.6914 × 10^−15^	0.4393	0.0036	0.0009
	Var	4.1382 × 10^−32^	7.2893 × 10^−30^	0.2594	0.0001	1.2328 × 10^−6^
*f*_2_	Min	0	2.2204 × 10^−16^	0.5852	0	0
	Max	0	2.6645 × 10^−14^	3.4157	0	0
	Ave	0	7.3053 × 10^−15^	1.7169	0	0
	Var	0	7.5842 × 10^−29^	1.2711	0	0
*f_3_*	Min	0	1.6653 × 10^−16^	0.0910	0	0
	Max	0	3.9351 × 10^−12^	2.0767	0	0
	Ave	0	5.8736 × 10^−13^	1.1458	0	0
	Var	0	1.6012 × 10^−24^	0.4273	0	0
*f*_4_	Min	0	5.5511 × 10^−17^	0.4008	0	0
	Max	0	6.8778 × 10^−14^	3.9791	3.3307 × 10^−16^	0
	Ave	0	1.5071 × 10^−14^	1.5279	6.6613 × 10^−17^	0
	Var	0	4.8923 × 10^−28^	1.4034	1.3559 × 10^−32^	0
*f*_5_	Min	0	2.2380 × 10^−17^	0.1529	0	8.5105 × 10^−5^
	Max	1.4374 × 10^−15^	9.8969 × 10^−15^	5.2062	0	0.0072
	Ave	1.4374 × 10^−16^	1.3184 × 10^−15^	2.3982	0	0.0015
	Var	2.0660 × 10^−31^	9.2626 × 10^−30^	2.5784	0	4.1821 × 10^−6^
*f*_6_	Min	1.3839 × 10^−87^	1.2181 × 10^−17^	0.2978	1.3839 × 10^−87^	0.0001
	Max	1.2738 × 10^−21^	1.3241 × 10^−15^	2.2916	1.3839 × 10^−87^	0.0554
	Ave	1.3301 × 10^−22^	4.7342 × 10^−16^	0.9435	1.3839 × 10^−87^	0.0258
	Var	1.6096 × 10^−43^	2.2174 × 10^−31^	0.4555	5.5373 × 10^−206^	0.0005
*f*_7_	Min	1.9671 × 10^−17^	6.8577 × 10^−15^	0.2525	0.0002	0.0002
	Max	0.0005	1.8490 × 10^−11^	15.7426	7.1992	0.0056
	Ave	9.1750 × 10^−5^	2.2728 × 10^−12^	5.7119	1.5894	0.0025
	Var	2.3620 × 10^−8^	3.2677 × 10^−23^	36.2206	7.2811	3.1310 × 10^−6^
*f*_8_	Min	3.2519 × 10^−27^	7.0257 × 10^−15^	0.8962	0.0064	0.0001
	Max	5.7350 × 10^−6^	2.4108 × 10^−12^	26.2582	39.3529	0.0368
	Ave	9.2305 × 10^−7^	5.0263 × 10^−13^	7.5532	5.2897	0.0104
	Var	4.0001 × 10^−12^	6.7474 × 10^−25^	52.2655	1.5027 × 10^2^	0.0002
*f*_9_	Min	1.3498 × 10^−31^	2.5846 × 10^−16^	0.2671	1.3498 × 10^−31^	0.0002
	Max	1.9689 × 10^−14^	7.3082 × 10^−14^	4.1046	1.3498 × 10^−31^	0.0099
	Ave	2.9178 × 10^−15^	1.6250 × 10^−14^	1.8569	1.3498 × 10^−31^	0.0033
	Var	4.3618 × 10^−29^	5.1105 × 10^−28^	0.9865	0	1.0867 × 10^−5^
*f*_10_	Min	1.7336 × 10^−38^	2.2204 × 10^−16^	0.0014	4.8795 × 10^−50^	5.0354 × 10^−54^
	Max	2.4876 × 10^−29^	2.0117 × 10^−13^	0.2567	1.6616 × 10^−10^	6.4181 × 10^−41^
	Ave	2.6360 × 10^−30^	2.5902 × 10^−14^	0.0544	1.6789 × 10^−11^	7.4082 × 10^−42^
	Var	6.1178 × 10^−59^	3.8412 × 10^−27^	0.0057	2.7550 × 10^−21^	4.0702 × 10^−82^
*f*_11(*D*=2)_	Min	8.8818 × 10^−16^	5.5532 × 10^−9^	0.4659	8.8818 × 10^−16^	8.8818 × 10^−16^
	Max	4.4409 × 10^−15^	5.5989 × 10^−8^	2.7931	2.5799	8.8818 × 10^−16^
	Ave	1.2434 × 10^−15^	1.8217 × 10^−8^	1.7149	0.2580	8.8818 × 10^−16^
	Var	1.2622 × 10^−30^	3.0345 × 10^−16^	0.5155	0.6656	0
*f*_12__(*D*=2)_	Min	0	0	0.0089	0	0
	Max	0.0074	0.0099	0.0150	0.0395	0.0085
	Ave	0.0015	0.0045	0.0115	0.0145	0.0016
	Var	9.7247 × 10^−6^	1.6088 × 10^−5^	4.6345 × 10^−6^	1.8438 × 10^−4^	1.1916 × 10^−5^
*f*_13__(*D*=2)_	Min	0	0	2.5027	0	0
	Max	0.9950	0.9950	5.5228	1.9899	0
	Ave	0.4869	0.0995	4.4264	0.3980	0
	Var	0.2644	0.0990	1.1062	0.4840	0
*f*_14(*D*=2)_	Min	9.4793 × 10^−39^	3.1793 × 10^−18^	0.0239	3.2958 × 10^−78^	1.0019 × 10^−57^
	Max	8.9804 × 10^−31^	1.4926 × 10^−16^	0.7788	1.8724 × 10^−19^	1.0246 × 10^−40^
	Ave	1.0017 × 10^−31^	5.2384 × 10^−17^	0.1987	1.8724 × 10^−20^	1.0247 × 10^−41^
	Var	7.9344 × 10^−62^	3.3914 × 10^−33^	0.0521	3.5060 × 10^−39^	1.0497 × 10^−81^

**Table 3:** Comparison of results for high dimension functions.

Function	Metric	VSCSA	CSA	CS	MFO	SCA
*f*_11(*D*=30)_	Min	2.2797	3.2397	16.3819	10.0741	0.6109
	Max	5.1136	6.0112	17.7649	16.5201	7.2651
	Ave	3.7778	4.6759	17.1658	13.7082	2.8626
	Var	0.6331	0.9435	0.2970	4.7736	3.6873
*f*_12(*D*=30)_	Min	0.0528	0.1364	1.2198	0.0300	0.0139
	Max	0.3587	0.3208	1.4571	0.5775	0.7466
	Ave	0.1378	0.2198	1.3653	0.2246	0.3989
	Var	0.0070	0.0056	0.0058	0.0388	0.0677
*f*_13(*D*=30)_	Min	1.1286 × 10^2^	1.3475 × 10^2^	1.4140 × 10^3^	1.5609 × 10^2^	1.1746 × 10^2^
	Max	2.5911 × 10^2^	2.0885 × 10^2^	2.0780 × 10^3^	9.2546 × 10^2^	2.5009 × 10^2^
	Ave	1.7758 × 10^2^	1.6834 × 10^2^	1.7499 × 10^3^	3.2263 × 10^2^	1.8308 × 10^2^
	Var	1.7198 × 10^3^	7.5248 × 10^2^	4.3256 × 10^4^	6.8344 × 10^4^	2.4061 × 10^3^
*f*_14(*D*=30)_	Min	0.1507	0.8730	1.1071 × 10^3^	2.5817	0.8212
	Max	0.4330	2.8546	1.8329 × 10^3^	8.0147 × 10^2^	59.5845
	Ave	0.2653	1.8225	1.5994 × 10^3^	3.4198 × 10^2^	16.5211
	Var	0.0075	0.4599	5.5006 × 10^4^	9.3346 × 10^4^	2.8519 × 10^2^
*f*_11(*D*=60)_	Min	4.1431	4.1958	16.7884	14.5856	5.0868
	Max	6.6470	5.7192	18.2277	18.1983	10.4431
	Ave	5.1825	4.8548	17.6613	16.7067	7.8758
	Var	0.6236	0.4106	0.2953	1.7958	3.1980
*f*_12(*D*=60)_	Min	0.2101	0.3262	1.7140	1.0847	0.0276
	Max	0.3411	0.6327	2.1414	1.4313	1.1034
	Ave	0.2524	0.4977	1.8979	1.2450	0.8115
	Var	0.0021	0.0087	0.0125	0.0143	0.1153
*f*_13(*D*=60)_	Min	3.3309 × 10^2^	3.7876 × 10^2^	3.6370 × 10^3^	8.6975 × 10^2^	2.1715 × 10^2^
	Max	6.2218 × 10^2^	6.1118 × 10^2^	5.3243 × 10^3^	3.9577 × 10^3^	7.7275 × 10^2^
	Ave	5.0434 × 10^2^	4.7404 × 10^2^	4.3379 × 10^3^	2.0584 × 10^3^	4.8009 × 10^2^
	Var	9.9127 × 10^3^	6.2803 × 10^3^	4.1675 × 10^5^	1.1259 × 10^6^	3.6050 × 10^4^
*f*_14(*D*=60)_	Min	2.7331	17.9029	2.6427 × 10^3^	2.9806 × 10^2^	1.2890 × 10^2^
	Max	5.5279	27.1171	4.5444 × 10^3^	1.5615 × 10^3^	4.8950 × 10^2^
	Ave	4.0482	21.1575	3.7431 × 10^3^	9.2456 × 10^2^	2.4631 × 10^2^
	Var	0.8931	8.1825	3.7021 × 10^5^	1.8256 × 10^5^	1.6048 × 10^4^
*f*_11(*D*=200)_	Min	4.9721	5.4329	16.4399	18.5124	8.4967
	Max	6.4728	6.2622	18.2605	18.9722	11.7968
	Ave	5.5735	5.7298	17.6394	18.7693	9.9552
	Var	0.2370	0.0481	0.2890	0.0161	1.2549
*f*_12(D=200)_	Min	0.5730	0.8061	3.6256	3.9739	1.1261
	Max	0.6674	0.9795	5.1979	4.8149	2.0141
	Ave	0.6197	0.9020	4.4142	4.2953	1.5740
	Var	0.0013	0.0035	0.1957	0.0626	0.0719
*f*_13(D=200)_	Min	1.8775 × 10^3^	1.9453 × 10^3^	1.5111 × 10^4^	1.5274 × 10^4^	1.7142 × 10^3^
	Max	2.5561 × 10^3^	2.3118 × 10^3^	1.8056 × 10^4^	1.7675 × 10^4^	4.6564 × 10^3^
	Ave	2.2061 × 10^3^	2.1896 × 10^3^	1.6534 × 10^4^	1.6121 × 10^4^	3.2054 × 10^3^
	Var	3.4431 × 10^4^	1.1518 × 10^4^	1.1715 × 10^6^	9.4756 × 10^5^	1.0841 × 10^6^
*f*_14(D=200)_	Min	42.8433	1.5256 × 10^2^	1.0562 × 10^4^	1.2646 × 10^4^	1.3509 × 10^3^
	Max	62.5081	2.0567 × 10^2^	1.6804 × 10^4^	1.5405 × 10^4^	5.0878 × 10^3^
	Ave	52.1370	1.8448 × 10^2^	1.3558 × 10^4^	1.4005 × 10^4^	3.1410 × 10^3^
	Var	33.5172	3.3378 × 10^2^	3.1386 × 10^6^	6.1661 × 10^5^	1.8390 × 10^6^

**Table 4:** Comparison of the Wilcoxon rank sum test results.

Function	CSA	CS	MFO	SCA
*f*_1_	0.00033	0.00018	0.00131	0.00018
*f*_2_	6.39 × 10^−5^	6.39 × 10^−5^	N	N
*f*_3_	6.39 × 10^−5^	6.39 × 10^−5^	N	N
*f*_4_	6.39 × 10^−5^	6.39 × 10^−5^	0.07758	N
*f*_5_	0.00219	0.00018	0.00023	0.00018
*f*_6_	0.00018	0.00018	0.00221	0.00018
*f*_7_	0.00283	0.00018	0.00033	0.00033
*f*_8_	0.27304	0.00018	0.00018	0.00018
*f*_9_	0.00443	0.00017	0.00597	0.00017
*f*_10_	0.00018	0.00018	0.79134	0.00018
*f*_11(*D*=2)_	0.00009	0.00009	1.00000	0.36812
*f*_12(*D*=2)_	0.01903	0.00013	0.01914	0.88154
*f*_13(*D*=2)_	0.87766	0.00015	0.60255	0.01429
*f*_14(*D*=2)_	0.00018	0.00018	0.00283	0.00018
*f*_11(*D*=30)_	0.07566	0.00018	0.00018	0.06402
*f*_12(*D*=30)_	0.01133	0.00018	0.67758	0.03121
*f*_13(*D*=30)_	0.73373	0.00018	0.18588	0.79134
*f*_14(*D*=30)_	0.00018	0.00018	0.00018	0.00018
*f*_11(*D*=60)_	0.57075	0.00018	0.00018	0.00283
*f*_12(*D*=60)_	0.00033	0.00018	0.00018	0.00283
*f*_13(*D*=60)_	0.42736	0.00018	0.00018	0.62318
*f*_14(*D*=60)_	0.00018	0.00018	0.00018	0.00018
*f*_11(*D*=20__0)_	0.14047	0.00018	0.00018	0.00018
*f*_12(D=200)_	0.00018	0.00018	0.00018	0.00018
*f*_13(D=200)_	0.85011	0.00018	0.00018	0.02113
*f*_14(D=200)_	0.00018	0.00018	0.00018	0.00018

**Table 8:** Results of three bar truss problem.

Algorithm	Min	Max	Std	Avg
WHO	263.8958433765	263.8958433765	1.2710574865 × 10^−13^	263.8958433765
PSO	263.8958433827	263.8960409745	5.3917161119 × 10^−5^	263.8959010895
GA	263.8958919373	263.9970875475	0.0252055577	263.9095296976
AEFA	265.1001279647	280.9534461900	4.0558625686	271.8733092380
FA	263.8958477145	263.8989975836	8.8455344984 × 10^−4^	263.8964634153
GSA	263.8968857660	264.1972851298	0.0948941056	264.0059193538
HHO	263.8959528570	264.0672685182	0.0467621287	263.9419743129
MVO	263.8958747019	263.9000377233	9.8601397499 × 10^−4^	263.8967256362
WOA	263.8959383525	265.6916186134	0.5029074306	264.3105859277
SSA	263.8958435096	263.8998220362	7.2678747873 × 10^−4^	263.8962415757
GWO	263.8959818300	263.9028435626	0.0014371714	263.8975822284
CSA	263.8958433765	263.8958433765	6.4741204424 × 10^−12^	263.8958433765
VSCSA	263.8958433765	263.9145156687	0.0037434952	263.8981466437

**Table 9:** Results of the gear train design problem.

Algorithm	Min	Max	Std	Avg
CS	2.7008571489 × 10^−12^	8.7008339998 × 10^−9^	2.5469034697 × 10^−9^	2.5277681200 × 10^−9^
FPA	2.3078157333 × 10^−11^	1.3616491391 × 10^−9^	5.1819924289 × 10^−10^	5.5155436237 × 10^−10^
FSA	1.0935663792 × 10^−9^	4.4677248806 × 10^−7^	8.5620977463 × 10^−8^	4.7845971457 × 10^−8^
SA	2.3078157333 × 10^−11^	1.3616491391 × 10^−9^	4.8777877665 × 10^−10^	6.1683323242 × 10^−10^
SCA	3.6358329757 × 10^−9^	2.0768133383 × 10^−1^	4.9002989331 × 10^−2^	1.6613443644 × 10^−2^
CSA	2.7008571489 × 10^−12^	2.3576406580 × 10^−9^	5.5363138249 × 10^−10^	2.7032649321 × 10^−10^
VSCSA	2.7008571489 × 10^−12^	2.7264505977 × 10^−8^	7.3324954585 × 10^−9^	4.4138792095 × 10^−9^

## Data Availability

The data used to support the findings of this study are included in this article.
